# A Radical-Free Approach to Teeth Whitening

**DOI:** 10.3390/dj9120148

**Published:** 2021-12-09

**Authors:** Mauro Pascolutti, Dileusa de Oliveira

**Affiliations:** Hismile Research Centre, 225-229 Burleigh Connection Rd., Burleigh Waters 4220, Australia; dileusa@hismileresearch.com

**Keywords:** dental bleaching, tooth whitening, phthalimidoperoxycaproic acid, dental erosion, microhardness, safety

## Abstract

Background: Traditional bleaching agents based on hydrogen peroxide (HP) or carbamide peroxide (CP) have adverse soft and hard tissue effects. Objectives: This study tested a novel formulation of phthalimidoperoxycaproic acid (PAP) with additives to optimise its safety and effectiveness. Methods: A novel gel (PAP+) was formulated. Laboratory studies assessed effects of six 10-minute exposures to PAP+ vs. commercial CP and HP gels, using surface profilometry and microhardness. The effectiveness of PAP+ in vitro against complex polyphenol stains on enamel was compared to 6% HP. Results: Unlike HP gels, PAP+ gel did not erode enamel. Unlike both CP and HP gels, PAP+ gel did not reduce the surface microhardness of enamel. PAP+ gel on used on polyphenol stains was superior to 6% HP. In this model, six repeated 10-minute treatments with PAP+ gel could improve the shade by approximately eight VITA^®^ Bleachedguide shades. Conclusions: These laboratory results support the safety and effectiveness of this new PAP formula and its use as an alternative to CP and HP with superior safety and effectiveness.

## 1. Introduction

Over the past decade, vital tooth bleaching (also known as tooth whitening) has become a popular procedure. Typical products used for at-home dental bleaching use as active ingredients either hydrogen peroxide (HP) [1] or its adduct carbamide peroxide (CP) [2]. The latter produces 35% of its weight as HP on contact with water. Different HP and CP gels are used currently for at-home and in-office dental bleaching according to jurisdictional regulations. The effects of HP and CP as bleaching agents improve with longer application times and greater concentrations of available hydrogen peroxide.

Several factors limit the usefulness of HP and CP in vital tooth bleaching, including their stability and their adverse effects on oral hard and soft tissues. Extended and repeated application can lead to oral mucosal irritation as well as dentinal hypersensitivity, and in some cases morphological and chemical changes to the enamel, including erosion and reduced surface microhardness [3,4,5]. Professional application (in-chair) protocols which involve the use of gingival barriers and soft tissue isolation can control the oral environment to reduce or prevent soft tissue irritation but cannot mitigate against adverse effects on enamel [6].

In recent years, a range of inexpensive home-bleaching products have become available through online vendors or over the counter (OTC). Many of these OTC products are used without professional workup or clinical supervision. Concerns for the safety of the teeth and oral soft tissues relate to the low pH of such products (which is intended to maintain their shelf life) [7], sub-optimal binding agents [8] and a lack of gingival protection [6].

In the search for bleaching agents that may be alternatives to HP or CP, there has been recent interest in using organic peroxides, such as phthalimidoperoxycaproic acid (PAP) as the active ingredient. PAP is available industrially as EURECO™ HC-L17™ (Solvay, Brussels, Belgium), which is a stable aqueous suspension of PAP crystals. PAP has a number of desirable safety characteristics, being considered non-toxic to humans, and it is readily biodegradable. The EURECO formulation when used at 83% for industrial clothes bleaching has been classified as non-corrosive to the skin and non-irritant.

In a recent laboratory study using a gel containing PAP, there was a reduction in enamel microhardness, and an etching effect was seen on the bleached enamel [9]. Such changes likely reflect an acidic pH and a non-optimal formulation. The current report describes studies using a novel formulation of PAP (designated as PAP+) that was designed to overcome these issues, with the intention of creating an efficient and safe whitening product suitable for the OTC market.

Traditional dental bleaching using HP or CP relies on free radicals, which oxidise organic pigments (chromogens). As these are converted into simpler or different structures, their optical properties change. The generation of different radical species from HP varies according to the pH and the method of activation [3]. Free radicals are unstable because they have an unpaired electron. To become stable, they will react with conjugated systems of unsaturated organic compounds. This breaks down the chromogens into simpler molecules in a redox reaction. The smaller reaction products produced from the oxidation process are less able to absorb light; hence, their colour is less intense [10,11].

**Figure 1:**
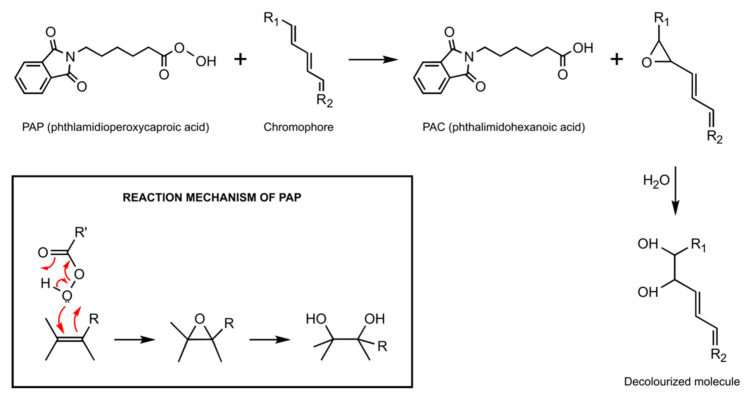
Mechanism of action of PAP on chromogens.

When using PAP, oxidation reactions also occur, which decolourise chromogens. The process involves epoxidation of molecules containing conjugated double bonds (Figure 1). This reaction occurs without the formation of free radicals. This is an important point since the free radicals are believed to be the primary cause of tooth sensitivity and gingival irritation during conventional tooth bleaching with HP and CP [12].

**Figure 2:**
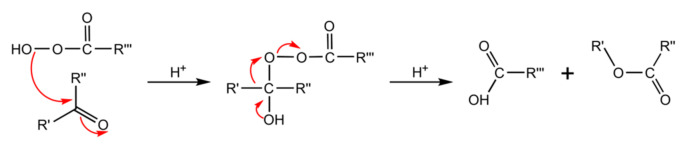
Mechanism of Baeyer-Villiger oxidation.

A range of molecules can serve as chromogens and cause intrinsic discolouration of vital teeth. There is a range of the reactions whereby PAP could alter chromogens. For example, in addition to the pathway presented in Figure 1, PAP can also react with ketones through a Baeyer-Villiger oxidation reaction (Figure 2).

A common chromogen in extrinsic tooth discolouration is polyphenols. These organic molecules are found abundantly in various coloured foods and beverages (including tea and red wine). They can be oxidised by peroxyacids to quinones and then potentially undergo further rearrangement reactions.

For the present study, the improved PAP product formulation included several additives not used with previous PAP dental bleaching products. Hydroxyapatite as a nano-sized powder was included to ensure saturation with apatite mineral, to prevent mineral loss and enamel softening. Potassium citrate was included, with the available potassium ions providing a desensitising effect for any exposed dentine or root surfaces. Using potassium citrate rather than the more common potassium nitrate was a deliberate choice, to establish a citrate buffer to maintain the pH at the desirable near-neutral pH range over time. Using this in combination with hydroxyapatite is designed to prevent any chelation of calcium from dental enamel.

The novel formulation also included an ammonium acryloyldimethyltaurate copolymer (Aristoflex AVC) as a binding agent. This was used to avoid the unwanted side effects of bio-adhesive polymers such as Carbopol on dental enamel that have been shown previously [8]. Including this binding agent into the bleaching gel formulation does not alter the effectiveness of bleaching.

In recent years, the effectiveness of PAP as a tooth-whitening ingredient has been investigated in a double-blind placebo-controlled clinical trial [13]. This showed significant bleaching effects after a single treatment, with no dental hypersensitivity or oral mucosal irritation. A more recent laboratory study published in 2019 compared a PAP-based gel against a conventional HP gel. While both had similar bleaching effects on bovine teeth, surface morphology and hardness measurements of the bleached teeth revealed that the HP gel caused some reduction in surface microhardness, while the PAP-based gel did not affect the integrity of enamel [14].

Based on this background, the present study was undertaken to explore the efficacy and safety of a novel PAP bleaching gel (Hismile™ PAP+) through (a) in vitro tests to assess enamel erosion and surface microhardness; (b) an in vitro assessment of bleaching effectiveness.

## 2. Materials and Methods

### 2.1. Enamel Erosion and Hardness Tests

PAP+ gel (Hismile Pty Ltd., Burleigh Waters, Qld, Australia) was compared with three experimental whitening gels with 6% HP (propylene glycol, glycerine, aqua, hydrogen peroxide, carbomer, carboxymethyl cellulose, triethanolamine, polyvinylpyrrolidone, mentha piperita oil, menthol), 35% HP (dual formula—1: hydrogen peroxide, glycerine, propylene glycol, aqua, triethanolamine, carbomer, carboxymethyl cellulose, polyvinylpyrrolidone, mentha piperita oil, menthol; 2: aqua, glycerine, propylene glycol, carbomer, sodium hydroxide, ferrous gluconate) and 35% CP (glycerine, urea peroxide, propylene glycol, xylitol, carbomer, mentha piperita oil, triethanolamine, aqua, ascorbic acid, eugenol, camellia sinensis leaf extract).

PAP+ gel was prepared from EURECO™ HC L17 by combining this with a vehicle of glycerine (as a humectant) mixed with acryloyldimethyltaurate copolymer (Aristoflex AVC) (Clariant International Pty Ltd., Muttenz, Switzerland) and polyvinyl pyrrolidone (PVP) (Plasdone K-29/32) (Ashland Inc, Wilmington, DE, USA). PVP is a pharmaceutical-grade, linear homopolymer of n-vinyl-2-pyrrolidone. PVP is bio-adhesive, readily soluble in water and solvents, physiologically inert, non-ionic, non-toxic, temperature-dependent and pH stable and is used in many bleaching gels. Other ingredients included potassium citrate (Jungbunzlauer Suisse AG, Basel, Switzerland), nano-hydroxyapatite (FLUIDNOVA, Portugal), titanium mica (BASF Colors & Effects GmbH, Ludwigshafen, Germany) saccharin sodium and peppermint oil. During manufacture, the final pH of the bleaching gel was adjusted to 6.5–7.0.

Extracted teeth removed for orthodontic reasons were collected by Intertek Clinical Research Services from private dental practices (Human Tissue Authority, license no. 12169, licence holder: ITS Testing Services UK Ltd.). Teeth were stored in 0.1% thymol immediately post extraction until use in the study. The teeth were used to prepare enamel slabs from the intact coronal enamel (4 mm × 4 mm). These slabs were then embedded in cylindrical moulds in epoxy resin (EpoxiCure2, Buehler, Lake Buff, IL, USA) and machine-polished using a polishing machine (Saphir 550, Unitron ATM, Mammellzen, Germany) to a final grade of 400 grit, to give a standardised flat surface. Six samples of enamel were prepared for each of the 4 treatment groups (6% HP, 35% HP, 25% CP and PAP+).

The baseline surface features of the samples were recording using a calibrated surface profilometer (Profilm 3D, Filmetrics, KLA Corp., San Diego, CA, USA). This unit uses white light interferometry (WLI) to measure surface profiles and roughness to an accuracy of 0.05 µm. The baseline surface hardness (expressed in Vickers hardness numbers (VHN)) was recorded using a microhardness tester (Tukon 1202, Wilson Hardness, Frankfort, IL, USA). Three surface microhardness measurements were measured for each sample under a 50 g load. Data sets were assessed for normality, and differences between groups were compared using analysis of variance.

Reference areas were formed in the enamel samples by covering half of each slab with tape in order to provide a baseline reference area for post-treatment surface profilometry assessments. The enamel surfaces were then moistened with distilled water prior to applying the bleaching gel. The influence of six 10 min consecutive applications of the assigned treatment on enamel was assessed. Approximately 0.5 g of bleaching gel was applied to the enamel using a cotton bud in a gentle wiping motion, ensuring the entire surface was evenly covered. After being left in place for 10 min, the gel was rinsed off with deionised water and surface-blotted lightly to remove excess moisture from the surface before the next application of gel. No saliva immersions were undertaken between treatments, to create a worst-case scenario.

After 6 consecutive treatment applications were performed, the surface profilometer was used to measure the erosive loss of the enamel, by comparing the treated areas with the reference areas that were protected from the treatment. The post-treatment Vickers surface microhardness was also measured. Data sets were assessed for normality, and differences between groups were compared using analysis of variance (Minitab18).

### 2.2. Bleaching Effectiveness In Vitro

A total of 30 human enamel slabs (5 mm × 5 mm) were embedded into acrylic spectrophotometer cuvettes using poly(methyl) methacrylate resin. The enamel surfaces of these samples were not polished or abraded, but instead were etched lightly using 1% HCl for 1 min in order to facilitate the binding of external stains, and then rinsed with water. To neutralise fully any residues of the acid, the blocks were immersed in a saturated sodium carbonate solution for 30 s then rinsed once again.

A staining solution was prepared that contained tryptone soy broth (TSB), instant tea, instant coffee, mucin type II, ferric chloride, red wine and deionised water. This broth was poured into the trough of a staining rig and kept in an incubator at 50 °C. The enamel samples were attached by wires to the staining rig. The samples were rotated continuously in and out of the staining broth at 1 rpm, such that all blocks were completely submerged at the lowest point of rotation. Blocks were removed periodically to assess stain uptake using a calibrated spectrophotometer (CM-700d, Konica Minolta Sensing Inc., Wayne, NJ, USA), tracking the change in L* over time. Once the staining had reached the darker end of the VITA^®^ Bleachedguide, all samples were removed. The samples with staining values closest to the lower range of the VITA^®^ Bleachedguide (i.e., darker than A 3.5) were then assigned randomly to one of five treatment groups (*n* = 6 per group for 2 groups). The baseline colour parameters (L*, a*, b*) of the stained enamel samples were measured using the spectrophotometer.

Each stained sample was then subjected to six consecutive applications of the assigned treatment—either the novel PAP gel or a 6% HP gel as positive control. The colour of each sample (L*, a*, b*) was measured before and after the sequence of 6 treatment applications. This measurement was done using 4 orientations, and the mean value from the 4 colour measurements was then used for analysis. A digital photograph was also taken at baseline and after the last treatment, for reference purposes. These photographs were not used for analysis.

Colour data were recorded directly into a ColourCalc Excel spreadsheet. The following formula was used to calculate the delta E for each treatment: ΔE = √((ΔL*)^2^ + (Δa*)^2^ + (Δb*)^2^). Delta E is a measure of the total change, with larger delta E values representing an increased bleaching effect. All spreadsheet formulae were subjected to a 10% randomised cell formula check, which were signed off by the allocated data checkers.

Prior to this part of the study, the number of delta E units required to move between the shades of the VITA^®^ Bleachedguide were calculated. These data were used to convert the total colour change achieved by each treatment into an equivalent number of VITA^®^ Bleachedguide shade unit changes.

Minitab version 18 software was used to calculate descriptive statistics for the total colour change data (delta E) comparisons. Data sets were assessed for normality, and changes caused by each treatment were assessed using a 2-sample *t* test.

## 3. Results

### 3.1. Enamel Erosion and Hardness Tests

The effects of 6 × 10 min applications of bleaching gels on erosion of the enamel followed two distinct patterns (Table 1). There was no enamel erosion seen with either 35% CP or with PAP+. Enamel surface loss from erosion (i.e., step defects) occurred in four of the six samples in each of the 6% HP and 35% HP groups. The extent of erosion in these groups was an average of 0.114 mm (SD 0.098) and 0.097 mm (SD 0.078), respectively. All data sets had Gaussian distributions. While erosion was 17.5% greater between 6% HP and 35% HP, this difference did not reach the threshold for statistical significance (two-tailed *p* value of 0.8229).

**Figure 3:**
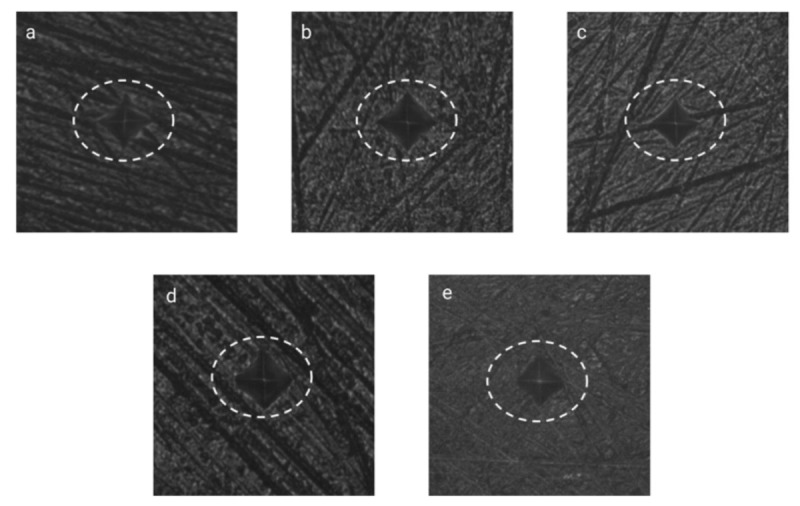
(**a**) Baseline SMH VK indents; (**b**) 6% HP post-treatment SMH VK indents; (**c**) 35% HP post-treatment SMH VK indents; (**d**) 35% CP post-treatment SMH VK indents; (**e**) PAP+ post-treatment SMH VK indents.

The microhardness results after six 10 min treatments also showed two distinct patterns (Table 1). For the PAP group, the Vickers surface microhardness increased after treatment (12.9 ± 11.7), and this change was significantly different from the other three groups (*p* < 0.001). All three commercial bleaching products caused a reduction in surface microhardness, with the 35% HP gel being ranked the worst in this regard (−94.28 ± 27.09), followed by 6% HP (−62.22 ± 19.52) and then by 35% CP (−55.3 ± 24.6), with no significant difference between the latter two products. In Figure 3, examples of baseline and post-treatment SMH VK indents of the four types of treatments are reported.

### 3.2. Bleaching Effectiveness In Vitro

**Figure 4:**
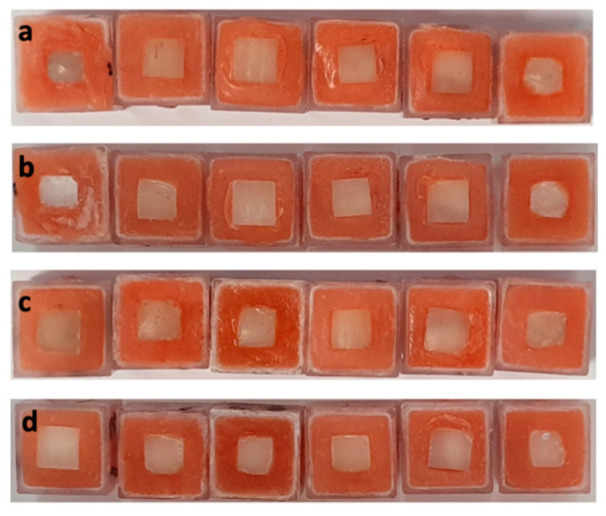
(**a**,**c**) Pre-treatment enamel discs; (**b**) 6% HP post-treatment enamel discs; (**d**) PAP+ post-treatment enamel discs. The images are intended for illustration purposes only.

The 6% HP gel used as a positive control gave a change in shade guide units (DSGU) of 4.86 ± 2.32, while the novel PAP+ gel caused an improvement of 8.13 ± 2.82, which was significantly greater in magnitude (two-tailed *p* value of 0.0110). All data sets had Gaussian distributions. Comparing the two (Table 2), the effect of PAP+ was greater than that of 6% HP by 70%. In other words, to gain the bleaching effect of two 10 min applications of PAP+ gel would require six 10 min treatments with 6% HP. The whitening achieved by the various treatments is shown in Figure 4.

## 4. Discussion

Overall, the results of this study provide insights into the safety and effectiveness of a novel bleaching gel based on PAP that has been formulated to address known problems with HP, CP and earlier PAP products.

The inclusion of hydroxyapatite and a citrate buffer to keep the PAP bleaching gel product at a similar pH value as normal resting saliva (pH 6.5–7.0) were together intended to prevent enamel surface loss from dental erosion and a reduction in surface microhardness. Past studies have shown that enamel erosion and mineral loss are worse when bleaching gels have a low pH and no bio-available calcium [15]. It is common for commercial HP-based products to have a low pH as this extends their shelf life. On the other hand, because of ammonia generation from the degradation of urea, carbamide peroxide-based gels tend to generate a higher pH when used and thus are less likely to cause enamel erosion [16]. The present findings are consistent with this since CP did not cause erosion. Moreover, the novel PAP gel did not cause any measurable enamel erosion. This finding suggests that the inclusion of hydroxyapatite and the presence of an efficient citrate buffering system that is able to maintain a near-neutral pH during treatment can preserve the enamel surface.

The same considerations follow through to the issue of surface microhardness. Several in vitro studies have reported that changes in microhardness are directly correlated to the degradation of the inorganic and organic components of the tooth surface [17,18,19], mostly due to the actions of free radicals. The present results for HP and CP causing reduced surface microhardness are consistent with prior studies. Of interest, the novel PAP gel caused a small increase in enamel microhardness. Such changes are consistent with previous observations of topically applied bio-available hydroxyapatite in dental products [20,21,22].

In phase 2 of the study, the laboratory assessment of changes in enamel slabs stained with a complex mixture of polyphenols showed that the PAP formulation was superior to the commercial 6% HP gel that was used as a positive control, by some 70% overall, in terms of shade guide unit changes. This particular test is clinically relevant because polyphenols are common forms of extrinsic stains on teeth. Moreover, they can be difficult to decolourise using free radicals as they have inherent antioxidant activity because of their molecular structure. The superior effectiveness and speed of action of the PAP product when compared to 6% HP as a point of comparison is noteworthy. The bleaching action of two 10 min applications with PAP+ gel was equivalent to six 10 min treatments with a typical 6% HP gel.

The positive performance of the novel PAP+ gel adds to previous evidence from in vitro and clinical studies supporting the use of PAP in bleaching gels as a safe and effective alternative in OTC products to HP and CP [13,14].

Future studies are needed to address a number of questions, including the ability of this treatment approach to treat stains that are typically resistant to HP or CP. Clinical studies with follow-up periods and larger cohort sizes would also be informative. Furthermore, morphological visualisation such as SEM (scanning electron microscopy) would be necessary to evaluate the morphological change of enamel topography when exposed to PAP+ formula.

The present in vitro studies were carried out at a single pH value and without any bleaching activation aids. Additional studies investigating the effect of PAP+ at different pH as well as in combination with bleaching accelerators (chemical activators or light irradiation devices) would be beneficial in providing a full overview of this new teeth-whitening formula.

## 5. Conclusions

In this study, a novel bleaching formulation based on phthalimidoperoxycaproic acid was used, with modifications designed to improve the effectiveness and safety, particularly in relation to effects on dental enamel and gingival soft tissues. Laboratory investigations revealed that that PAP+ gel does not erode enamel or reduce the surface microhardness of enamel, which stands in contrast to enamel loss and softening seen with commercial HP and CP bleaching gels. A laboratory assessment of the effectiveness of the PAP+ gel on polyphenol stains showed enhanced performance when compared to a 6% hydrogen peroxide gel. In this model, repeated 10 min treatments with PAP+ gel could improve the shade by approximately eight VITA^®^ Bleachedguide shades.

Within the limitation of this current study, it was concluded that the above results support the safety and effectiveness of this new PAP-based formula (PAP+) and its use as an alternative to CP and HP with superior safety and effectiveness. The inclusion of hydroxyapatite and potassium citrate proved to be essential to maintain a near-neutral pH during treatment and preserve the enamel surface.

## Figures and Tables

**Table 1:** Post-treatment changes in enamel erosion and surface microhardness (SMH).

Treatment	Enamel Erosion (mm)	Change in SMH	Mean Enamel Erosion (mm)	Mean Change in SMH	SMH SD
6% HP	0.183	−42.67	0.114	−62.22	19.52
	0.00	−54.33			
	0.115	−45.00			
	0.140	−68.67			
	0.00	−67.33			
	0.245	−95.33			
35% HP	0.149	−97.00	0.097	−94.28	27.09
	0.121	−75.00			
	0.00	−80.67			
	0.124	−147.33			
	0.186	−79.00			
	0.00	−86.67			
35% CP	0.00	−65.7	0.00	−55.3	24.6
	0.00	−100.7			
	0.00	−47.3			
	0.00	−35.0			
	0.00	−39.3			
	0.00	−43.7			
PAP+	0.00	−7.0	0.00	12.9	11.7
	0.00	14			
	0.00	7.3			
	0.00	23.0			
	0.00	25.0			
	0.00	15.3			

**Table 2:** Mean Post Treatment Changes in VITA^®^ Bleachedguide Shades.

Treatment	Timepoint	VITA Bleached Guide Shades Changes	SD
6% HP	1st treatment	1.97	0.76
PAP+		3.86	1.41
6% HP	2nd treatment	2.82	1.09
PAP+		5.79	2.20
6% HP	3rd treatment	3.95	1.50
PAP+		6.59	2.13
6% HP	4th treatment	4.44	2.03
PAP+		6.59	2.46
6% HP	5th treatment	4.44	2.58
PAP+		7.32	2.79
6% HP	6th treatment	4.86	2.32
PAP+		8.13	2.82

## Data Availability

Not applicable.

## References

[1] Fearon J. (2007). Tooth whitening: Concepts and controversies. J. Ir. Dent. Assoc..

[2] Viscio D., Gaffar A., Fakhry-Smith S., Xu T. (2000). Present and future technologies of tooth whitening. Compend. Contin. Educ. Dent. Suppl..

[3] Rodríguez-Martínez J., Valiente M., Sánchez-Martín M.J. (2019). Tooth whitening: From the established treatments to novel approaches to prevent side effects. J. Esthet. Rest. Dent..

[4] Tredwin C.J., Naik S., Lewis N.J., Scully C. (2006). Hydrogen peroxide tooth-whitening (bleaching) products: Review of adverse effects and safety issues. Br. Dent. J..

[5] Sulieman M.A.M. (2008). An overview of tooth-bleaching techniques: Chemistry, safety and efficacy. Periodontology 2000.

[6] Briso A.L.F., Rahal V., Gallinari M.O., Soares D.G., de Souza Costa C.A., Perdigão J. (2016). Complications from the use of peroxides. Tooth Whitening: An Evidence-Based Perspective.

[7] Jurema A.L.B., de Souza M.Y., Torres C.R.G., Borges A.B., Caneppele T.M.F. (2018). Effect of pH on whitening efficacy of 35% hydrogen peroxide and enamel microhardness. J. Esthet. Rest. Dent..

[8] Gouveia T.H.N., de Souza D.F.S., Aguiar F.H.B., Ambrosano G.M.B., Lima D.A.N.L. (2020). Effect of ammonium acryloyldimethyltaurate copolymer on the physical and chemical properties of bleached dental enamel. Clin. Oral Investig..

[9] Greenwall-Cohen J., Francois P., Silikas N., Greenwall L., Le Goff S., Attal J.P. (2019). The safety and efficacy of ‘over the counter’ bleaching products in the UK. Br. Dent. J..

[10] Watts A., Addy M. (2001). Tooth discolouration and staining: A review of the literature. Br. Dent. J..

[11] Joiner A. (2006). The bleaching of teeth: A review of the literature. J. Dent..

[12] Kwon S.R., Wertz P.W. (2015). Review of the mechanism of tooth whitening. J. Esthet. Rest. Dent..

[13] Bizhang M., Domin J., Danesh G., Zimmer S. (2017). Effectiveness of a new non-hydrogen peroxide bleaching agent after single use—A double-blind placebo-controlled short-term study. J. Appl. Oral Sci..

[14] Qin J., Zeng L., Min W., Tan L., Lv M., Chen Y. (2019). A bio-safety tooth-whitening composite gels with novel phthalimide peroxy caproic acid. Compos. Commun..

[15] Rodrigues F.T., Serro A.P., Polido M., Ramalho A., Figueiredo-Pina C.G. (2017). Effect of bleaching teeth with hydrogen peroxide on the morphology, hydrophilicity, and mechanical and tribological properties of the enamel. Wear.

[16] Potočnik I., Kosec L., Gašperšič D. (2000). Effect of 10% carbamide peroxide bleaching gel on enamel microhardness, microstructure, and mineral content. J. Endod..

[17] Pinto C.F., de Oliveira R., Cavalli V., Giannini M. (2004). Peroxide bleaching agent effects on enamel surface microhardness, roughness and morphology. Braz. Oral Res..

[18] Redha O., Strange A., Maeva A., Sambrook R., Mordan N., McDonald A., Bozec L. (2019). Impact of a carbamide peroxide whitening agent dentinal collagen. J. Dent. Res..

[19] Wijetunga C.L., Otsuki M., Abdou A., Luong M.N., Qi F., Tagami J. (2021). The effect of in-office bleaching materials with different pH on the surface topography of bovine enamel. Dent. Mater. J..

[20] Ebadifar A., Nomani M., Fatemi S.A. (2017). Effect of nano-hydroxyapatite toothpaste on microhardness ofartificial carious lesions created on extracted teeth. J. Dent. Res. Dent. Clin. Dent. Prospect..

[21] Esteves-Oliveira M., Santos N.M., Meyer-Lueckel H., Wierichs R.J., Rodrigues J.A. (2017). Caries-preventing effect of anti-erosivive and nano-hydroxyapatite-containing toothpaste in vitro. Clin. Oral Investig..

[22] Sudradjat H., Meyer F., Loza K., Epple M., Enax J. (2020). In Vivo Effects of a Hydroxyapatite-Based Oral Care Gel on the Calcium and Phosphorus Levels of Dental Plaque. Eur. J. Dent..

